# Examining polarizing and non-polarizing filters for road sports

**DOI:** 10.3389/fspor.2023.1236473

**Published:** 2023-08-04

**Authors:** Luca Mercatelli

**Affiliations:** CNR-INO National Institute of Optics, Firenze, Italy

**Keywords:** polarization, sunglasses, road sports, road luminance, polarizing filters

## Abstract

The use of sunglasses and polarized sunglasses is common in all aspects of life and is very popular in outdoor athletic activities. However, the choice of athletes regarding their sunglasses is often not dictated by the performance ensured by one model rather than another but by other factors such as look or wearability due also to the lack of technical data on cataloguess. A conscious choice of filters to use, also according to road and weather conditions, supported by quantitative data, would instead allow athletes to improve their visual comfort and sport experience. The transmission spectra of 10 pairs of sports sunglasses (five polarized and five not polarized) were measured and related to the road luminance for different sun positions in both horizontal and vertical polarizations. The luminous transmission factor was calculated, and the luminance quantities while wearing the glasses were defined and calculated for all cases. A survey was submitted to a group of athletes to collect their impressions and relate them to the measurement results. The pairs of polarized and non-polarized sports sunglasses showed similar transmission factors, so their ability to mitigate outdoor light is almost the same—the main difference lies in the polarized light transmitted. Outdoor measurements showed that the light reflected by the road has a substantial polarized horizontal component. Polarized sunglasses block much of the light reflected by the road, resulting in a darker appearance of the road and reducing the glare of the scene in bright sunlight. The decrease in road luminance increases the contrast discrimination of other objects in the scene, which reflect sunlight in a non-polarized way (e.g., non-flat surfaces). The survey demonstrates that interviewed athletes prefer polarized sunglasses for these aformentioned reasons. This study highlights the advantages of polarizing sunglasses for athletic activities on roads because the road surface often reflects polarized light depending on the position of the sun.

## Introduction

Sunglasses are usually worn by athletes during sports activities on the road, as they protect against particles or insects directed into the eyes, sometimes at high speed (1, 2). Nowadays, almost all sunglasses for sports activities are equipped with plastic lenses with UV-blocking filters, which are a good barrier against this part of the sun spectrum, which is dangerous for the anterior part of the eye (3, 4). Among all the different models, sunglasses for sports activities can be equipped with polarized or non-polarized lenses (5).

Polarization is one of the properties of transverse waves: in linearly polarized radiation, the electric field oscillates along the same direction, and is perpendicular to the direction of propagation, for example, in laser light (6).

Most light sources emit unpolarized light, but there are several ways in which light in nature can be polarized, including reflection, absorption, and scattering (7).

In natural light, the electric field oscillates randomly in the plane perpendicular to the direction of propagation, but when light meets a reflecting dielectric surface, such as water, it undergoes a process of partial polarization, i.e., the component along the direction of the reflection plane undergoes less attenuation than that in the perpendicular one (8). In particular, when total reflection occurs on the interface separating two media with different refractive indexes, at the Brewster angle, the reflected light is completely linearly polarized along the direction of the reflection plane: for this reason, as an example, the glare on the sea surface is horizontally polarized (9). The degree of horizontal polarization decreases when the incident angle is smaller than the Brewster angle (also called the “polarizing angle”).

Polarizing filters are used to isolate the polarized component along a specific direction because the human eye cannot distinguish polarized light from non-polarized light (except under specific conditions) (10, 11). In the case of reflection on a water surface, the glare can be reduced by wearing glasses with vertical polarizing filters, which heavily reduce or block horizontally polarized radiation.

In fact, the fishing sector widely uses polarized lenses because, in addition to being a good protection against UV rays, they reduce the reflections of the sun on the water, allowing for better vision.

When total reflection occurs, the interface from one medium to another behaves as a mirror, and the polarized reflection induces a disability glare in the observer, which reduces the ability to perceive the visual information needed for a particular activity. Disability glare is caused by light scattered within the eye, which causes a haze of veiling luminance that decreases contrast and reduces visibility (12, 13). One of the first studies on polarization and vision evaluated the effect on visual acuity with and without polarized sunglasses in the presence of glare: polarized ones significantly improved visual performance during a glare condition (14). Polarized lenses are also important while driving: when a person is heading toward the direction of the sun and wearing polarized lenses, the glare by reflections from wet roads is reduced. Thus, the driver easily recognizes details on the road that could be hidden in the event of glare (15).

Classic Fresnel reflection and polarization described with the Brewster angle refer to specular reflection from a smooth surface separating two media with different refractive indexes (e.g., air–water), but if we consider the reflection of light caused by road asphalt, the analysis is much more complicated since there is a mixture of specular and diffuse reflection, resulting in an articulated lobe. However, as polarized light is related to the specular reflection, the worst cases of polarized glare in the case of asphalt will occur when the sun is in front of the observer, placed in the direction of the specular reflection. Wet conditions will amplify the specular reflections, as may oil, etc. (16), but our work focuses on dry conditions, as outdoor sports activities are performed more often in good weather.

Light diffused by the rough and dry surface of asphalt is a mixture of reflected and diffused radiation, as stated above, and the specular reflection is not easily recognizable. Therefore, this will lead to a discomfort glare. In this case, the glare does not significantly reduce the ability to see information needed for a specific activity (disability glare) but is distracting and uncomfortable for the observer as it interferes with the perception of visual information. In this case, glare is that annoying phenomenon that covers the entire field of vision and mainly causes reduced visibility, fatigue, eye irritation, and color distortion. This case is analyzed in our work, as road-polarized radiance is measured and related to using polarized/non-polarized sunglasses.

On the other hand, polarized lenses also have some limitations, such as the vision of strains on some materials (e.g., windshields and metals), due to the mechanical stress they are subjected during the manufacturing process. These phenomena are more evident, for example, when the incident light is already polarized and the observer uses a polarizing filter.

Studies have also shown that visual acuity for viewing moving objects declines when lighting falls below a specific level (17). This finding implies that polarized lenses could have an effect on visual acuity in sports in low light conditions due to the reduced level of illumination, but this is beyond the scope of this work as sports activities on the road are usually performed in daylight. Moreover, in sports performed under natural sunlight, some tinted lenses are preferred. Furthermore, polarized lenses could be tinted. However, for this study, to avoid other variables, we used neutral (gray) lenses (18). In this research, we compared the performance of polarized and non-polarized filters used for road sports, measuring their spectral characteristics and relating them to the reflection characteristics of the road itself. This approach provides a quantitative evaluation that can be used, among other parameters, to choose a particular sports eyewear.

## Methods

The methods used to evaluate the performances of polarized sunglasses during sport activities on the road are divided into three sections, namely, (1) spectrophotometric transmittance measurements of the sunglasses under controlled conditions in the laboratory, (2) measurement of the road radiance and its polarization characteristics as a function of sun position, and (3) a brief survey on a group of athletes after a 1–2-week test of each polarized and non-polarized sunglasses. In this work, five types of sunglasses for road sports (four different brands) were identified, and two samples were considered for every type, namely, the polarized and non-polarized versions.

### Spectral measurements on sunglasses

Spectral transmittance of all 10 sunglasses was measured in horizontal, vertical, and no polarization using a spectrophotometer (Lambda 900, PerkinElmer). Transmittance spectra were acquired for polarized and non-polarized glasses both in polarized and non-polarized light to verify that (a) the spectral transmittance of polarized and non-polarized light is similar for sunglasses of the same type, (b) the transmittance of the horizontally polarized light is nearly zero (within experimental errors, 4%) for polarizing sunglasses, and (c) the differences between the transmittance of horizontal and vertical polarized light are equal within experimental uncertainties for non-polarized sunglasses (also, in this arrangement, errors in spectral transmittance are within 4%).

An internal depolarizer was used in all the measurements to eliminate the partial polarization introduced by the mirrors and gratings of the instrument. After the depolarizer, a motorized polarizing filter introduced the desired and controlled polarization axis, while the reference beam was non-polarized. Measurements were performed every 5 nm in the range of 280–850 nm, with a bandpass of 2 nm. Considering that the different sunglasses could differ in light transmission, to verify and normalize all the quantities, the luminous transmission factor *τ_v_* (in percentage value) was calculated for every sample according to (19)

(1) $$\tau_{V}=\frac{\int_{380\;\mathrm{nm}}^{780\;\mathrm{nm}}\tau_{F}\left(\lambda \right)\cdot S_{D65}\left(\lambda \right)\cdot V\left(\lambda \right)\;d\lambda }{\int_{380\;\mathrm{nm}}^{780\;\mathrm{nm}}S_{D65}\left(\lambda \right)\cdot V\left(\lambda \right)\;d\lambda }$$

where *S_D_*_65_(*λ*) is the solar spectrum, *V*(*λ*) is the photopic spectral sensitivity, and *τ_F_*(*λ*) is the spectral transmittance of the sunglass. The luminous transmission factor was also determined for polarized glasses in the condition of vertical polarization, as the polarizing filter blocks the horizontal component. The uncertainty on the luminous transmission factor is 4%.

### Road radiance measurements

**Figure 1:**
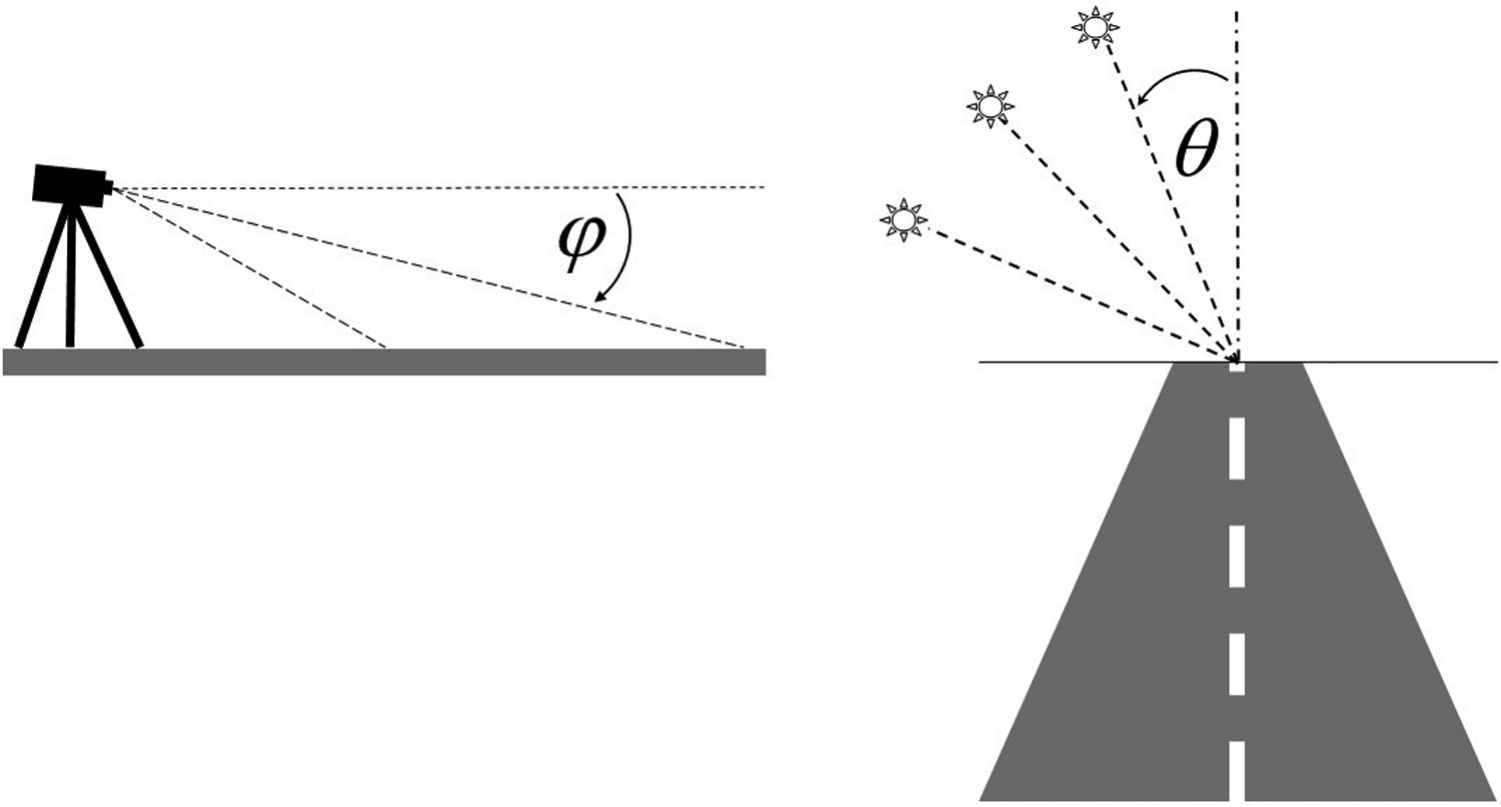
Measurements of the spectral radiance of the road, considering two pointing directions and four angles of the sun with respect to the road. The spectrometer is pointed on the road, and the direction makes an angle *φ* of 10° or 20° with the horizon direction. The sun is in front at angle *θ* with the road direction of 6.5°, 11°, 18°, and 22.5°.

The measurements of the road spectral radiance were performed on a sunny day using a fiber optic portable spectrometer (AvaSpec 2048, Avantes). The road section elected for measurement is flat and straight, 800 m long, and in dry conditions. Measurements were performed in June (*T* = 26°, *RH* = 57%). No rain had fallen for at least 1 week. The asphalt is more than 10 years old, and there is exclusively vehicular, non-heavy, and non-intense traffic. Thus, the road section represents most Italian roads used for daily sports training (run, road bike). The spectra were acquired every 1 nm in the range of 300–1,000 nm (bandwidth of 0.8 nm). A group of 10 athletes (triathletes) was asked to wear the glasses and indicate the point of the road where they usually look during a run or bike ride. The positions of the eyes (height) with respect to the road and the directions of sight were achieved, and a measurement set-up was prepared based on these data. The head of the spectrometer, equipped with a lens and a rotating polarizing filter (Melles Griot, Glan–Thompson polarizer, range 300–2,300 nm, extinction ratio: 5 × 10^−5^), was held on a tripod placed 1.40 m above the road plane and was pointed alternately with an angle *φ* of 10° or 20° below the direction of the horizon (Figure 1). The spectral transmittances of the lens and polarizing filter were measured using the spectrophotometer (Lambda 900, PerkinElmer), and the spectral response of the system was corrected for their transmittances. To equalize the measurement viewing angle of the spectrometer system with a standard 2° observer, a calibration was performed with a luminance meter (Minolta, mod. LS110), and a correction constant *C* was achieved. The measurements of road spectral radiance were acquired for both polarizations (horizontal and vertical) for both *φ* angles and with different sun positions with respect to the road. During measurements, the sun was positioned in front of the observer, forming angles *θ* of 6.5°, 11°, 18°, and 22.5° with the road direction (Figure 1); the height of the sun ranged between 30° and 65° above the horizon. Measurements were taken twice each time, moving from one polarization to the other and from one angle to the other, to evaluate reproducibility error: reproducibility is within instrumental errors, also taking into account errors derived from lens and polarization filter measurements (10%).

To calculate the luminance perceived by the subject while wearing the sunglasses, in the case of polarized or non-polarized sunglasses, the quantity was defined (in Cd/m^2^):

(2) $$LP_{n}=C\int_{380\;\mathrm{nm}}^{780\;\mathrm{nm}}R_{n}(\lambda )\cdot \tau_{n}(\lambda )\cdot V(\lambda )\;d\lambda$$

where *n* is alternately “H” (horizontal polarization), “V” (vertical polarization), or “NP” (no polarization); *R*(*λ*) is the spectral radiance of the road; and *τ*(*λ*) is the spectral transmittance of the sunglasses.

Equation (2) represents the luminance perceived wearing sunglasses, road radiance, filter transmission, and eye sensitivity and the calibration constant *C*. The uncertainty on the calculation of LP, derived from the different contributions of spectral measurements and calibration, is 15%.

In the case of polarized sunglasses, as their transmission of vertically polarized light is nearly equal to zero, we can conclude that LP_V_ ≅ 0.

### Survey

After conducting spectral measurements, the sunglasses were distributed to a group of 21 triathlon athletes in pairs. Each athlete received either polarized or non-polarized versions of the same sunglasses for them to wear during their daily training sessions for 7–14 days, alternating between the pairs of glasses. Every athlete usually trains 10–14 h/week on roads, running and cycling. A rotation system allowed all the athletes to try all five pairs of sunglasses. Labels regarding polarization were removed from the sunglasses, and every pair of glasses was randomly labeled as A and B to counterbalance the order in which they were worn. Athletes did not have access to the association table and were asked to collaborate and not to try to ascertain which sunglasses were polarized and which were not, although they could figure it out through simple experiments. The survey submitted to the athletes (answers were anonymous) comprised four questions. According to the association table, an answer scale of 1–9 was proposed, where 1 = best with polarized glasses, 5 = neutral, and 9 = best with non-polarized glasses. The questions included (1) Do you see more saturated colors with A or B glasses? (2) Do you see sharper objects with A glasses or B glasses? (3) Can you predict/see obstacles (potholes, etc.) on the road better with A glasses or B glasses? (4) Do you have a more comfortable vision in passages with different brightness of the road (e.g., light areas/shaded areas) with A or B glasses? The survey aimed to understand the vision comfort with polarized and non-polarized sunglasses. No statistical analysis was conducted, and all the answers obtained from the group of athletes were reported directly.

## Results

### Spectral measurements on sunglasses

**Figure 2:**
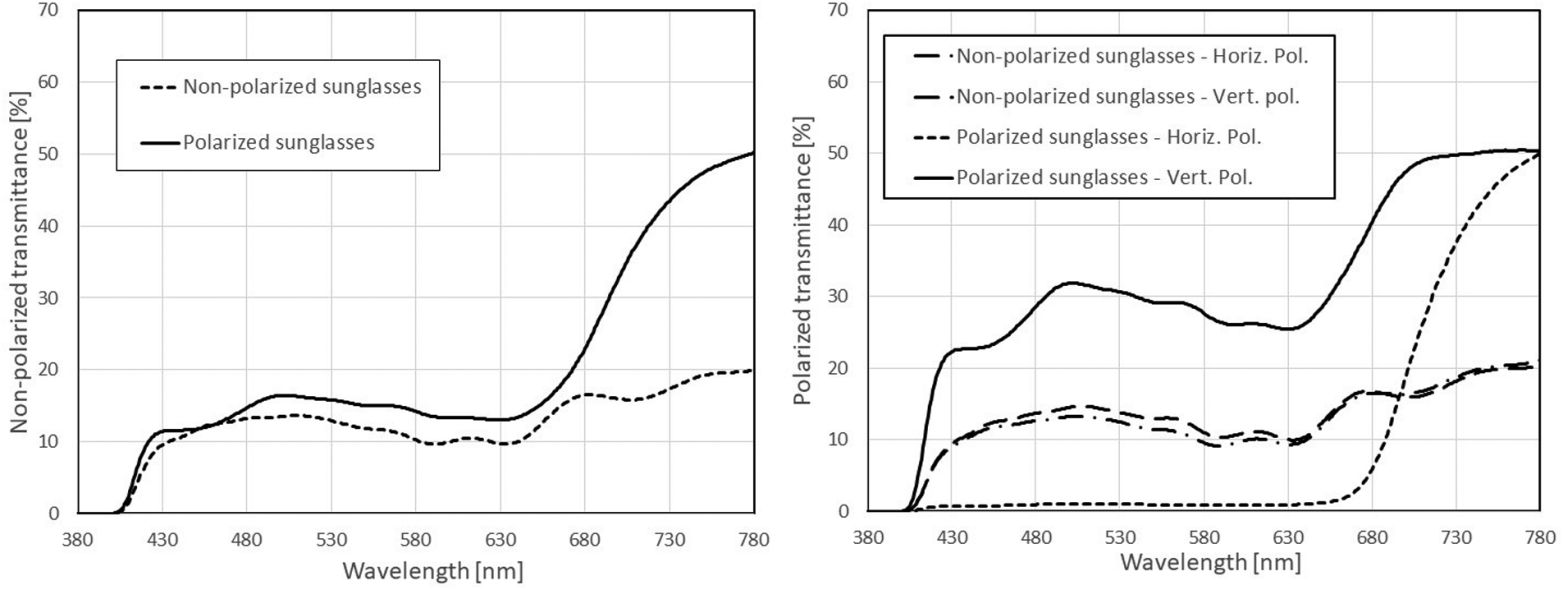
Spectral transmission of two pairs of sunglasses (same type) in non-polarized light (left): transmission of polarized and non-polarized sunglasses is similar in the range of vision while differing in the NIR range. Spectral transmission of the same pairs of sunglasses in polarized light (right). Transmission is high in vertical polarization and below 1% in horizontal polarization. Transmission of non-polarized sunglasses is constant in both polarizations within experimental errors.

Measurements showed that the spectral transmittances of the pairs of the same sunglass type were similar when tested in non-polarized light (Figure 2), considering that all lenses were gray and transmission factors of the sunglasses were comparable. When tested in polarized light, the non-polarized sunglasses showed similar transmission spectra in both vertical and horizontal polarizations, with differences within experimental errors, as expected. In contrast, the polarized sunglasses showed high transmittance in vertical polarization and very low transmittance (below 1%) in horizontal polarization. Thus, almost all horizontally polarized light was blocked.

### Measurements of road radiance and luminance perceived using sunglasses

The spectral polarized road radiance was measured for two directions and at different hours of the day to ensure that the angle of the sun changed with respect to the road. Not all the combinations were achieved as certain sky conditions did not allow the exact measurements of polarized quantities in some instances (e.g., glazes). Measurements showed that the horizontal component is higher than the vertical one. Their ratio, calculated as a mean of the measurements in the two pointing directions (10° and 20° below horizon), ranged from 1.3 to 2.6 (Table 1).

**Table 1:** Values, in Cd/m^2^, of the road polarized luminance as a function of the sun position.

*θ* angle Angle of the sun with respect to the road direction	*L*_H_ (*φ* = 10°)	*L*_V_ (*φ* = 10°)	*L*_H_ (*φ* = 20°)	*L*_V_ (*φ* = 20°)	*L*_H_^mean^	*L*_V_^mean^	$\frac{L_{H}^{\mathrm{mean}}}{L_{V}^{\mathrm{mean}}}$
6.5	6,710	2,680	6,690	2,460	6,700	2,570	2.6
11			4,490	2,380	4,490	2,380	1.9
18	7,910	4,980	6,950	6,300	7,430	5,640	1.3
22.5	7,240	4,980			7,240	4,980	1.5

The closer the sun to the road direction (specular reflection), the higher the horizontal component with respect to the vertical one, while for larger solar angles, light reaches the photometer primarily by diffuse rather than specular reflection. Values are rounded to the nearest tens, with greater measurement errors (10%).

**Figure 3:**
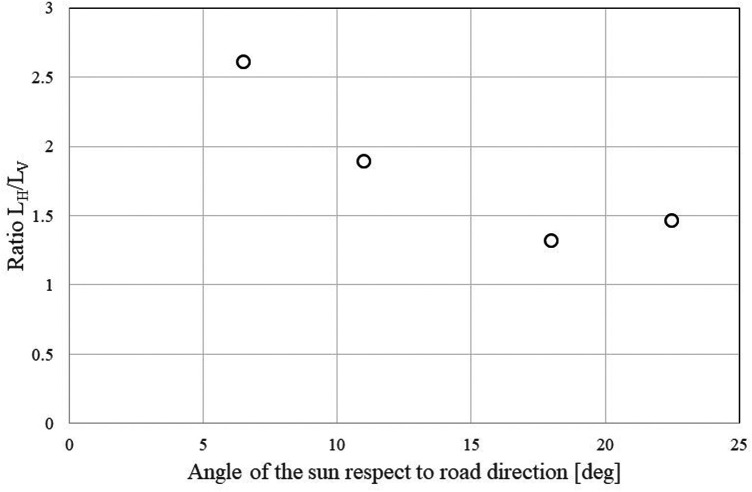
Ratio between horizontally and vertically polarized luminance of the road as a function of sun position with respect to the road. The zero angle represents the road direction.

When the sun is in front of the road (i.e., for smaller angles of the sun with respect to the road direction), the horizontal component is considerably higher than the vertical one. This observation means that the glare of the road is at maximum (Figure 3).

Polarized sunglasses block a significant amount of luminance from the road because light reflected from the road is mainly horizontally polarized. To compare luminance perceived with polarized and non-polarized sunglasses, differences in luminous transmittance of the glasses of the same type must be considered. Table 2 reports luminance perceived in different situations; in all the cases, the luminance perceived with polarized sunglasses is only a fraction of the one perceived with non-polarized sunglasses, with this fraction ranging from 3% to 16%. Considering that the luminance perceived with non-polarized sunglasses is 100%, the luminance perceived with polarized lenses will always be less than 20%. Thus, the road will appear one-fifth as luminous compared with how it appears using traditional sunglasses.

**Table 2:** Road luminance perceived with polarized and non-polarized sport glasses for different sun positions with respect to the road (*θ*) and different pointing angles (*φ*).

			Sunglasses				
			Type no. 1	Type no. 2	Type no. 3	Type no. 4	Type no. 5
		*τ_v_* (pol.)	14.8%	19.0%	13.9%	16.2%	19.6%
		*τ_v_* (non-pol.)	11.7%	19.2%	15.4%	15.5%	10.8%
*θ* angle: angle of the sun with respect to the road direction	6.5°	LP_V_ (*φ *= 10°)	61	48	21	116	61
		LP_NP_ (*φ *= 10°)	617	883	661	723	510
		**LP_V_/LP_NP_ (*φ*** = **10°)**	**8%**	**6%**	**4%**	**15%**	**7%**
		LP_V_ (*φ* = 20°)	65	52	23	116	66
		LP_NP_ (*φ* = 20°)	663	899	697	705	505
		**LP_V_/LP_NP_ (*φ*** = **20°)**	**8%**	**6%**	**4%**	**16%**	**7%**
	11°	LP_V_ (*φ* = 20°)	40	32	14	70	40
		LP_NP_ (*φ* = 20°)	576	634	474	479	367
		**LP_V_/LP_NP_ (*φ*** = **20°)**	**6%**	**5%**	**3%**	**14%**	**6%**
	18°	LP_V_ (*φ* = 10°)	73%	58	25	126	73
		LP_NP_ (*φ* = 10°)	842	1,200	901	912	692
		**LP_V_/LNP (*φ*** = **10°)**	**7%**	**5%**	**3%**	**13%**	**6%**
		LP_V_ (*φ* = 20°)	68	54	24	120	69
		LP_NP_ (*φ* = 20°)	955	1,307	1,007	1,018	734
		**LP_V_/LP_NP_ (*φ*** = **20°)**	**6%**	**4%**	**3%**	**11%**	**5%**
	22.5°	LP_V_ (*φ* = 10°)	66	52	23	114	66
		LP_NP_ (*φ* = 10°)	787	1,125	843	853	650
		**LP_V_/LP_NP_ (*φ*** = **10°)**	**7%**	**5%**	**3%**	**13%**	**6%**

The luminance perceived with polarized sunglasses is a fraction (3–16%) of the one perceived with non-polarized sport sunglasses. Bold indicates ratios of polarized/non-polarized.

### Survey

**Figure 4:**
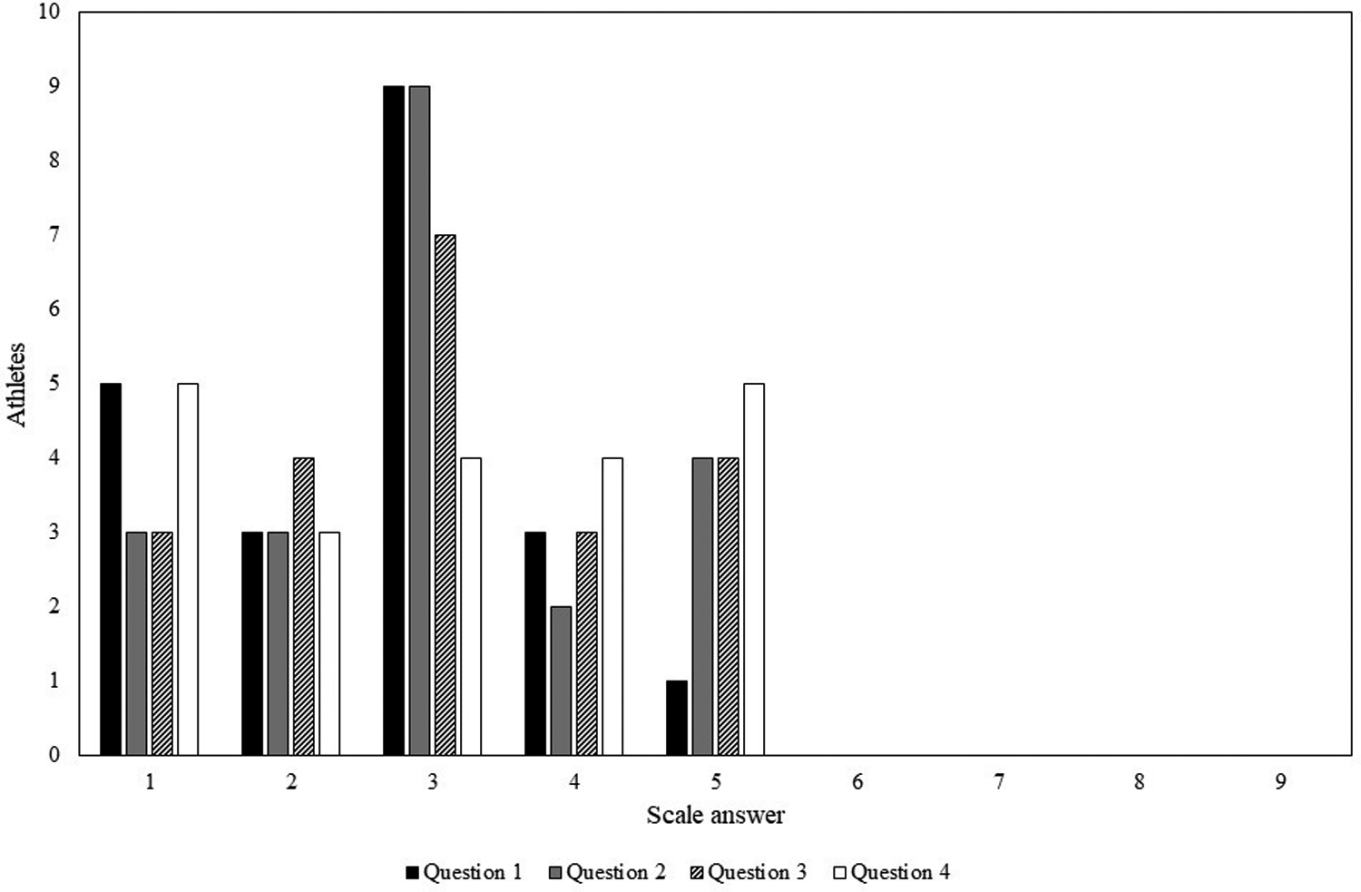
Answers to the questions (1) Do you see more saturated colors with non-polarized or polarized glasses? (2) Do you see sharper objects with non-polarized or polarized glasses? (3) Can you predict/see obstacles (potholes, etc.) on the road better with np glasses or p glasses? (4) Do you have a more comfortable vision in passages with different brightness of the road (e.g., light areas/shaded areas) with non-polarized or polarized ones? The scale 1–9 in the abscissa represents the degree of preference (1 = best polarized, 5 = neutral, and 9 = best non-polarized), and the ordinate represents the number of athletes. A rotation system allowed all athletes to try all sunglasses, with polarization labels removed, to avoid discrimination of polarized and non-polarized sunglasses.

The survey responses from the triathletes demonstrated that most athletes preferred polarized glasses, while only five or fewer athletes out of 21 maintained neutrality for every question. In particular, athletes found that polarized sunglasses were always moderately better than non-polarized ones because the answer with the most occurrences was number 3 (Figure 4). In question no. 4, responses were more constant as it concerns even more subjective evaluations than the other three questions. The survey pointed out that the athletes see more saturated colors, sharper objects, and discontinuities on the road using polarized sunglasses, and this may be because the road appears darker when wearing polarized sunglasses.

## Discussion

Polarized eyewear helps eliminate glare, especially in water sports or skiing, where glare partially inhibits vision (20, 21). Our findings suggest that using polarized eyewear, even during sports activities performed on the road (running, cycling), is beneficial for athletes. In fact, measurements on a typical training road show that the light reflected from the road is partially horizontally polarized: the more the sun in front of the athlete, the more polarized the reflection. Therefore, using polarized sunglasses eliminates much of the light reflected from the road, which makes it appear darker, potentially limiting the discomfort glare. Thus, the effect of polarized sunglasses can be extended to other contexts, in addition to those involving wet roads or reflections on liquid media (9), extending a quantitative evaluation to other fields. This work suggests a quantitative methodology supported by subjective interviews with athletes, an approach that we want to extend to a wider range of laboratory and road measurements in the future, extending it to other types of sunglasses (e.g., photochromatic).

## Conclusions

Sunglasses for road sports were chosen from brands and models that offered both polarized and non-polarized lens types. In total, 10 sunglasses were analyzed: five polarized and five non-polarized. Spectral transmittance of all sunglasses was measured in non-polarized light, and it was verified that the luminous transmission factor and spectral characteristics of the two versions (pol. and non-pol.) of the same sunglasses were similar. Four pairs of glasses had very small differences in transmission factor (0.2%–3.1%), while one pair had a more significant difference between the two versions (8.8%). Different brands did not show typical differences, as optical differences are attributable to the model more than the brand, mainly because different models of the same brand sometimes have lenses produced by different manufacturers. Measurements of the polarized luminance of a typical Italian secondary road (asphalt 10 years old, low traffic), which athletes use to train away from city traffic, showed that the horizontally polarized component is higher compared with the vertically polarized one. Their ratio goes from 1.3 to 2.6 when the sun is positioned in front of the road. Thus, the luminance perceived wearing polarized sunglasses, which blocks the horizontal component, is just a fraction of the one perceived with non-polarized ones: the fraction perceived goes from 3% to 16%, which means that the road appears darker when athletes wear polarized sunglasses. This finding, along with the fact that glare is removed, gives the athlete who wears polarized sunglasses better vision in terms of sharpness and recognition of details of the road, which may be crucial in sports like cycling. The survey submitted to the group of athletes evidenced that polarized sunglasses provided a better vision experience during road sports such as running or cycling, and measurements performed in this work can explain their answer in terms of road luminance and glare suppression.

## Data Availability

The raw data supporting the conclusions of this article will be made available by the authors without undue reservation.
